# Cognitive swarming in complex environments with attractor dynamics and oscillatory computing

**DOI:** 10.1007/s00422-020-00823-z

**Published:** 2020-03-31

**Authors:** Joseph D. Monaco, Grace M. Hwang, Kevin M. Schultz, Kechen Zhang

**Affiliations:** 1 Department of Biomedical Engineering, Johns Hopkins University School of Medicine, Baltimore, MD 21205 USA; 2 The Johns Hopkins University/Applied Physics Laboratory, Laurel, MD 20723 USA

**Keywords:** Swarming, Multi-robot groups, Place cells, Oscillations, Spatial navigation, Emergence

## Abstract

Neurobiological theories of spatial cognition developed with respect to recording data from relatively small and/or simplistic environments compared to animals’ natural habitats. It has been unclear how to extend theoretical models to large or complex spaces. Complementarily, in autonomous systems technology, applications have been growing for distributed control methods that scale to large numbers of low-footprint mobile platforms. Animals and many-robot groups must solve common problems of navigating complex and uncertain environments. Here, we introduce the NeuroSwarms control framework to investigate whether adaptive, autonomous swarm control of minimal artificial agents can be achieved by direct analogy to neural circuits of rodent spatial cognition. NeuroSwarms analogizes agents to neurons and swarming groups to recurrent networks. We implemented neuron-like agent interactions in which mutually visible agents operate as if they were reciprocally connected place cells in an attractor network. We attributed a phase state to agents to enable patterns of oscillatory synchronization similar to hippocampal models of theta-rhythmic (5–12 Hz) sequence generation. We demonstrate that multi-agent swarming and reward-approach dynamics can be expressed as a mobile form of Hebbian learning and that NeuroSwarms supports a single-entity paradigm that directly informs theoretical models of animal cognition. We present emergent behaviors including phase-organized rings and trajectory sequences that interact with environmental cues and geometry in large, fragmented mazes. Thus, NeuroSwarms is a model artificial spatial system that integrates autonomous control and theoretical neuroscience to potentially uncover common principles to advance both domains.

**Electronic supplementary material:**

The online version of this article (10.1007/s00422-020-00823-z) contains supplementary material, which is available to authorized users.

## Introduction

Rodent spatial cognition has been extensively studied in non-naturalistic environments such as linear or circular tracks, radial arm mazes, and T-mazes, or small open-field arenas such as squares or cylinders of approximately 1–2 $\hbox {m}^2$ area. Such conditions have allowed individual place fields of hippocampal pyramidal neurons (O’Keefe and Dostrovsky 1971) and the activity of other spatial cells (Knierim 2006; Moser et al. 2008; Savelli et al. 2008; Poulter et al. 2018; Wang et al. 2018) to be exquisitely controlled and analyzed, leading to a detailed neural coding account of distributed representations that subserve spatial learning, memory, and planning in mammals including humans (O’Keefe and Nadel 1978; Moser and Paulsen 2001; Knierim and Hamilton 2011; Monaco and Abbott 2011; Pfeiffer and Foster 2013; Hartley et al. 2014; Burgess 2014; Schiller et al. 2015; Foster 2017; Bellmund et al. 2018; Kunz et al. 2019). However, the multiplicity of Poisson-distributed hippocampal place fields exposed in larger environments (Fenton et al. 2008; Rich et al. 2014) and species differences in mapping 3-dimensional contexts (Yartsev and Ulanovsky 2013; Casali et al. 2019) suggest that large and/or complex environments are the next frontier in understanding spatial navigation.

Computational models of rodent spatial networks have typically emulated the restricted environments of experimental studies (for computational efficiency, ease of analysis, and compatibility with published data). Despite these limitations, recent theoretical results have demonstrated the importance of sensory and cortical feedback in stabilizing and shaping hippocampal place cell and entorhinal grid cell representations (Monaco et al. 2011; Poll et al. 2016; Rennó-Costa and Tort 2017; Ocko et al. 2018); this relationship has been supported by experimental approaches to the animals’ own active sensing behaviors such as lateral head scanning (Monaco et al. 2014; Yadav and Doreswamy 2017) and closed-loop control of orienting distal cues (Jayakumar et al. 2019). Additionally, extending theoretical frameworks such as attractor maps (Zhang 1996; Tsodyks 1999; Samsonovich and McNaughton 1997; Knierim and Zhang 2012) to large spaces has shown increased network capacities for computation and memory (Hedrick and Zhang 2016). Thus, extending theory to large or complex environments may require closed-loop integration of sensory information with internal spatial maps.

Complementary to animal studies, investigating artificial spatial systems comprising virtual and/or robotic mobile agents may help to elucidate spatial cognitive computations in naturalistic contexts (Hasselmo 2018; Tomov et al. 2018; Savelli and Knierim 2019; Gaussier et al. 2019). Artificial networks trained on path integration spontaneously produced grid cell-like patterns (Cueva and Wei 2018; Banino et al. 2018), suggesting a possible link to shared neurocomputational principles (n.b., Savelli and Knierim 2018). Additionally, the neural representations of the hippocampus and related structures have motivated several approaches to spatial mapping, planning, and navigation for robotic platforms (Milford et al. 2004; Cuperlier et al. 2007; Milford and Wyeth 2008; Barrera and Weitzenfeld 2008). These neuromimetic models have relied on the representations of place cells, head direction cells, border cells, and/or grid cells to drive spatial computations in support of single-platform robotic control (Milford et al. 2010; Tejera et al. 2018; Kreiser et al. 2018; Balaji et al. 2019; Gaussier et al. 2019). It has remained unclear how the spatiotemporal dynamics of these neural representations might reciprocally inform advances in autonomous control.

Biomimetic approaches have been applied to swarming problems, which require collective behaviors to accomplish spatially distributed tasks. One such approach, inspired by animal groups with oscillatory communication patterns, was generalized as the ‘swarmalators’ formalism (Iwasa and Tanaka 2010; O’Keeffe et al. 2017), in which an agent’s internal phase is governed by local Kuramoto synchronization and swarming attraction and repulsion are phase-coupled. However, swarmalator systems naturally relax to static states or simple cycling behaviors (ibid.). Thus, we investigated how the spatiotemporal dynamics of hippocampal circuits might drive useful exploratory or navigational behaviors in distributed groups of mobile oscillators via swarming. Hippocampal phenomena that have been theorized to support biological spatial cognition include (1) self-stabilizing activity patterns in attractor map networks and (2) temporal-phase organization relative to a global oscillation. We demonstrate that attractor dynamics and phase-based organization can be driven, in parallel, by a form of Hebbian learning modified to operate on, and indirectly control, inter-agent distances. While attractor dynamics have been demonstrated for low-level mechanical control (Nurzaman et al. 2015) and spatial mapping (Milford et al. 2004; Milford and Wyeth 2008), we show that attractor dynamics can also be recruited as a high-level navigational control. Further, our link from learning to swarming is a fast online process, unlike pre-trained or slowly adapting neural network controllers.

In this paper, we introduce the NeuroSwarms controller framework with analogies to neuroscience and an example implementation (Sect. 2). The following sections present emergent swarming behaviors in simulations of a fragmented and heterogeneous environment (Sect. 3.1), demonstrate NeuroSwarms operating in a single-entity paradigm that can be studied to provide insights into animal spatial cognition (Sect. 3.2), evaluate reward-approach behaviors in a large hairpin maze (Sect. 3.3), and discuss implications for autonomous systems design and spatial cognition in large, complex environments (Sect. 4). Thus, the neurodynamics of hippocampal function may reveal a path toward decentralized self-organization for future applications of autonomous swarming.

**Fig. 1:**
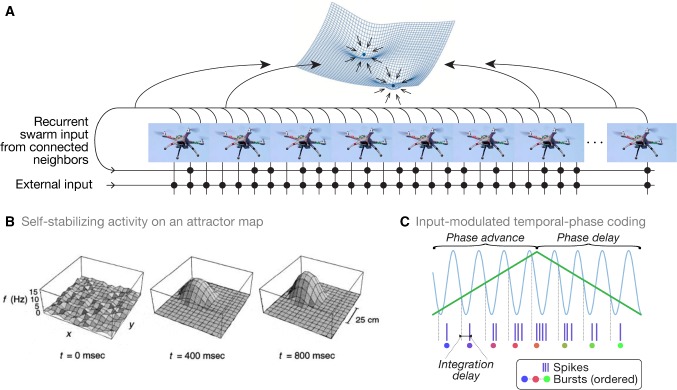
Conceptual schematic and theoretical neuroscientific inspiration for the NeuroSwarms controller. **a** An artificial spatial system of mobile virtual or robotic agents communicate over sparse recurrent channels (bottom) just as spatial neurons in biological neural circuits produce reverberating activity patterns that reflect energy minima in the dynamical state-space of the system (e.g., fixed-point attractors; top; adapted from Knierim and Zhang 2012). **b** Example simulation of the spatial self-organization of an activity bump on an attractor map. In an attractor map network, the environment is represented by a continuum of locations with overlapping place fields, leading to network connectivity that produces self-reinforcing spatial activity patterns. Adapted from Zhang (1996). **c** Schematic of a minimal model of temporal-phase coding in which an excitatory external input (green) is rhythmically modulated by a continuous inhibitory oscillation (blue) such as the hippocampal theta rhythm. Adapted from Monaco et al. (2019a) as permitted by the CC-BY 4.0 International License (creativecommons.org/licenses/by/4.0/) (color figure online)

## Models and methods

### Model analogy: swarms as spatial neuron circuits

Hippocampal place cells fire within a contiguous region of the animal’s local environment, or ‘place field’ (O’Keefe and Dostrovsky 1971). Our key realization was that an individual agent could be represented as a spatial neuron (e.g., a place cell) whose preferred location, or place field, indicates the agent’s desired position in the environment. It follows that a multi-agent group would be analogous to a neuronal network (e.g., the recurrently connected place cells of hippocampal subregion CA3). Connections between neurons may be characterized by the ‘synaptic weight’ that acts as a multiplicative gain on neuronal inputs. We further suppose that mutually visible agent pairs are reciprocally connected and that the distance between them maps to the symmetric synaptic weight of those connections. Consequently, relative agent motion with respect to environmental geometry corresponds to changes in connectivity and weights. Thus, a spatial configuration of the group constitutes an attractor map network (Zhang 1996; Tsodyks 1999; Samsonovich and McNaughton 1997) and relative motion (i.e., swarming) constitutes learning based on synaptic modification (Hebb 1949; Oja 1982). Figure 1a illustrates the analogy of a swarm of robotic platforms that form synapse-like connections and network-like collective behaviors, such as fixed-point attractors.

### Hippocampal mechanisms

#### Self-stabilizing attractor maps

Place fields are thought to collectively form cognitive maps (O’Keefe and Nadel 1978) that are stabilized (at least in part) via attractor dynamics, such as fixed points or continuous manifolds of the network energy surface, that drive activity toward low-dimensional spatial or task representations (Fig. 1b; Knierim and Zhang 2012). Attractor map models have shown that recurrent connectivity between place cells with nonlinear integration of inputs is nearly sufficient to achieve stable spatial attractors (Zhang 1996; Samsonovich and McNaughton 1997; Tsodyks 1999). For instance, a rate-based network following

1 $$\begin{aligned} \frac{\mathrm{d}r_i}{\mathrm{d}t} = -r_i + g\left( \sum _j J_{ij}r_j + I_i \right) \, , \end{aligned}$$

where $r_i$ is the rate of unit *i*, $I_i$ is the unit’s total input, and *g* is a sigmoidal nonlinearity, only further requires that the recurrent weights $J_{ij}$ encode the degree of place-field overlap between units (i.e., the strength of learned spatial associations). Such an encoding follows from a kernel function of field-center distances, e.g.,

2 $$\begin{aligned} J_{ij} := F({\mathbf {x}}_i - {\mathbf {x}}_j) = A \ \exp \left( -\frac{|{\mathbf {x}}_i-{\mathbf {x}}_j|^2}{\sigma ^2}\right) - B \, , \end{aligned}$$

where $\mathbf {x}_k$ is the field-center position of unit *k*, $\sigma$ is the Gaussian scale constant, and *A* and *B* determine the strength of local excitation vs. long-range inhibition, respectively. While this formulation violates Dale’s law, it illustrates the typical parsimony of attractor map models (Tsodyks 1999). A network constructed from (1) and (2) supports self-organization of its activity into a singular, contiguous ‘bump’ that emerges as the network relaxes (Fig. 1b; Zhang 1996). The activity bump can then respond to input changes due to, e.g., movement through the environment or internal processing. These conditions are encapsulated by the NeuroSwarms analogy (Sect. 2.1).

#### Oscillatory phase coding

Spatial activity in the rodent hippocampal formation is strongly modulated by the prominent theta (5–12 Hz) rhythm during active behaviors (Vanderwolf 1969; Buzsáki 2005). In place cells, this modulation produces ‘theta-phase precession,’ a monotonic advance in spike timing from late to early within each theta cycle that may support precise spatial coding and sequence learning (O’Keefe and Recce 1993; Jensen and Lisman 2000; Foster and Wilson 2007; Drieu et al. 2018). Recently, we discovered a novel class of spatial phase-coding neurons, termed ‘phaser cells,’ that were located in a major subcortical target of hippocampal output (Monaco et al. 2019a). In contrast to phase precession, we found that theta phase was symmetrically coupled to firing rate in phaser cell recordings, suggesting that an intrinsic phase-coding mechanism transforms spatial information into theta phase (ibid.). This phase-coding mechanism is consistent with a minimal model of input-driven oscillators (Fig. 1c), which we have implemented as Eq. (12) in our NeuroSwarms model (Sect. 2.4).

### Internal place fields for swarm control

There are two reasons why neural swarming control should decouple physical location from internal self-localization. First, the multiplicity of agents is a qualitative difference with brain circuits; every place cell in a biological network operates for the same agent (e.g., the rat). Given the analogy of agents to neurons (Fig. 1a; Sect. 2.1), an agent should have only one place field despite a rat having many. Further, an agent’s physical location depends on momentary circumstances of operating in the real (or simulated) world, but its place field should reflect intended locations. Second, experimental studies have compellingly demonstrated that path planning in hippocampal networks may rely on place-cell sequences that represent remote locations (Gupta et al. 2010; Pfeiffer and Foster 2013; Ólafsdóttir et al. 2018; Momennejad et al. 2018), indicating that internal representations are separable from an animal’s physical location. Thus, we assign cue-driven place preferences to each NeuroSwarms agent.

### NeuroSwarms: mobile oscillatory Hebbian learning

In this section, we develop an oscillatory learning model for a group of $N_s$ mobile agents according to our motivating analogy (Sect. 2.1; see Sect. 4). We emphasize that the NeuroSwarms framework encompasses the concept of swarming as learning as expressed above; the particular model that we present here is one example. Following the Gaussian attractor map kernel from (2), we explicitly relate a recurrent weight matrix ${\varvec{W}}\in {\mathbb {R}}^{N_s\times N_s}$, prior to learning-based updates, to swarm state via

3 $$\begin{aligned} W_{ij}=V_{ij}\exp (-D^2_{ij}/\sigma ^2), \end{aligned}$$

for inter-agent visibility ${\varvec{V}}\in \{0,1\}^{N_s\times N_s}$, inter-agent distances ${\varvec{D}}$, and spatial constant $\sigma$. To provide environmental interactions, we consider a minimal reward-approach mechanism for a set of $N_r$ reward coordinates that serve as attractive locations. Thus, we relate a feedforward weight matrix ${\varvec{W}}^r\in {\mathbb {R}}^{N_s\times N_r}$, prior to learning-based updates, to swarm state via

4 $$\begin{aligned} W^r_{ik}=V_{ik}^r\exp (-D^r_{ik}/\kappa ), \end{aligned}$$

for agent-reward visibility ${\varvec{V}}^r\in \{0,1\}^{N_s\times N_r}$, agent-reward distances ${\varvec{D}}^r$, and spatial constant $\kappa$. The reward weights are based on an exponential kernel to allow for long-range approach behaviors.

As inputs, we consider that each agent’s internal place field derives from the conjunction of sensory cue inputs related to a preferred location. We define $N_c$ sensory cues with inputs ${\varvec{c}}\in {\mathbb {R}}^{N_s\times N_c}$ following

5 $$\begin{aligned} \tau _c\dot{c}_{ik} = V^c_{ik}V^{c^*}_{ik} - c_{ik} \, , \end{aligned}$$

for cue *k*, agent-cue visibility ${\varvec{V}}^c\in \{0,1\}^{N_s\times N_c}$, fixed agent-cue preferences ${\varvec{V}}^{c^*}\in \{0,1\}^{N_s\times N_c}$, and integration time constant $\tau _c$. We define reward inputs ${\varvec{r}}\in {\mathbb {R}}^{N_s\times N_r}$ following

6 $$\begin{aligned} \tau _r{\dot{r}}_{ik} = V^r_{ik}-r_{ik} , \end{aligned}$$

for reward *k* and integration time constant $\tau _r$. Unlike sensory cues, all agents respond equally to rewards when visible. We define recurrent inputs ${\varvec{q}}\in {\mathbb {R}}^{N_s\times N_s}$,

7 $$\begin{aligned} \tau _q{\dot{q}}_{ij} = V_{ij}\cos (\theta _j-\theta _i) - q_{ij} \, , \end{aligned}$$

to agent *i* from agent *j* with integration time constant $\tau _q$ and internal phase ${\varvec{\theta }}$. We chose to implement the phase coupling of the recurrent swarming input in (7) as the cosine of phase differences between pairs of agents (cf. O’Keeffe et al. 2017). The cosine provides an even and circularly periodic function of phase similarity for synchrony-driven attraction (via positive input values) or repulsion (via negative input values) in conjunction with the learning process below.

We consider net inputs to each agent as gain-modulated and visibility-normalized quantities for sensory cue inputs,

8 $$\begin{aligned} I_{c_i} = \frac{g_c}{\sum _{k}V^c_{ik}}\sum _{k=1}^{N_c}c_{ik} , \end{aligned}$$

reward inputs,

9 $$\begin{aligned} I_{r_i} = \frac{g_r}{\sum _{k}V^r_{ik}}\sum _{k=1}^{N_r}W_{ik}^rr_{ik} , \end{aligned}$$

and recurrent swarming inputs,

10 $$\begin{aligned} I_{q_i} = \frac{g_s}{\sum _{j}V_{ij}}\sum _{j=1}^{N_s}W_{ij}q_{ij} , \end{aligned}$$

with the constraint that $g_c$, $g_r$, and $g_s$ sum to 1. For example, the net cue input $I_c$ in (8) is a temporally smoothed, weighted fraction of visible cues that are preferred by each agent. Thus, place-field size is determined by the relative cue richness of the environment: More cues will tend to increase heterogeneity and spatial selectivity. Because the net inputs are bounded in (8)–(10), we apply linear rectification rather than a saturating nonlinearity (cf. (1)) to calculate activation

11 $$\begin{aligned} {\varvec{p}} = \left[ I_c+I_r+I_q\right] _+ , \end{aligned}$$

which is the remaining component needed to compute Hebbian (or any two-factor) learning. Additionally, the model agents are phase-coupled via (7), and thus, we consider that the activation ${\varvec{p}}$ drives the internal phase state (see Sect. 4), e.g.,

12 $$\begin{aligned} {\dot{{\varvec{\theta }}}}={\varvec{\omega }}_0+\omega _I\,{\varvec{p}}, \end{aligned}$$

where $\omega _I$ sets the maximum increase in input-modulated angular frequency above the baseline frequency ${\varvec{\omega }}_0$. This mechanism, in which phase differences (7) drive activation (11) and synchronization (12), gives agents both place-cell-like spatial tuning (Sect. 2.2.1) and phaser-cell-like phase coding (Sect. 2.2.2).

The core of the NeuroSwarms controller comprises the learning-based updates to ${\varvec{W}}$ and ${\varvec{W}}^r$. A naïve Hebbian rule, such as $\hbox {d}W_{ij}=\eta p_i q_j$, would cause weights to grow unbounded and, therefore, inter-agent distances to converge to zero. Instead, we calculate updated swarming weights ${\varvec{W}}^\prime$ following

13 $$\begin{aligned} W^{\prime }_{ij} = W_{ij}+\Delta t\,\eta V_{ij}\,p_i(q_{ij}-p_iW_{ij}) , \end{aligned}$$

with simulation time step $\Delta t$ and learning rate $\eta$, which effectively normalizes connection strengths to each agent according to Oja’s rule (Oja 1982). Likewise, we calculate updated reward weights ${\varvec{W}}^{r\prime }$ following

14 $$\begin{aligned} W^{r\prime }_{ik}=W^r_{ik}+\Delta t\,\eta _r V^r_{ik}\,p_i(r_{ik}- \ p_iW^r_{ik}) . \end{aligned}$$

These weight update rules impose, via the implicit normalization, a baseline level of depression of the weights and, equivalently, a baseline level of repulsion between agents that counteracts the tendency to synchronize and converge at the same locations (see Sect. 4).

### Reward capture

In certain simulations, specified as having ‘capturable’ rewards, visited reward locations are remembered by an agent and the reward ceases to attract that agent. We implemented reward capture using the parameter $d_\mathrm{rad}$ (Table 1) and the reward visibility matrix ${\varvec{V}}^r$. For agent *i* and reward *k*, when the agent enters the contact radius, $D^r_{ik}\le d_\mathrm{rad}$, then we update $V^r_{ik}\leftarrow 0$ (multi-agent) or $V^r_{\cdot k}\leftarrow 0$ (single entity) and prevent subsequent updates. This causes agents to ignore the reward by forcing to zero the agent-reward weight (4), input (6), and weight updates (14).

**Fig. 2:**
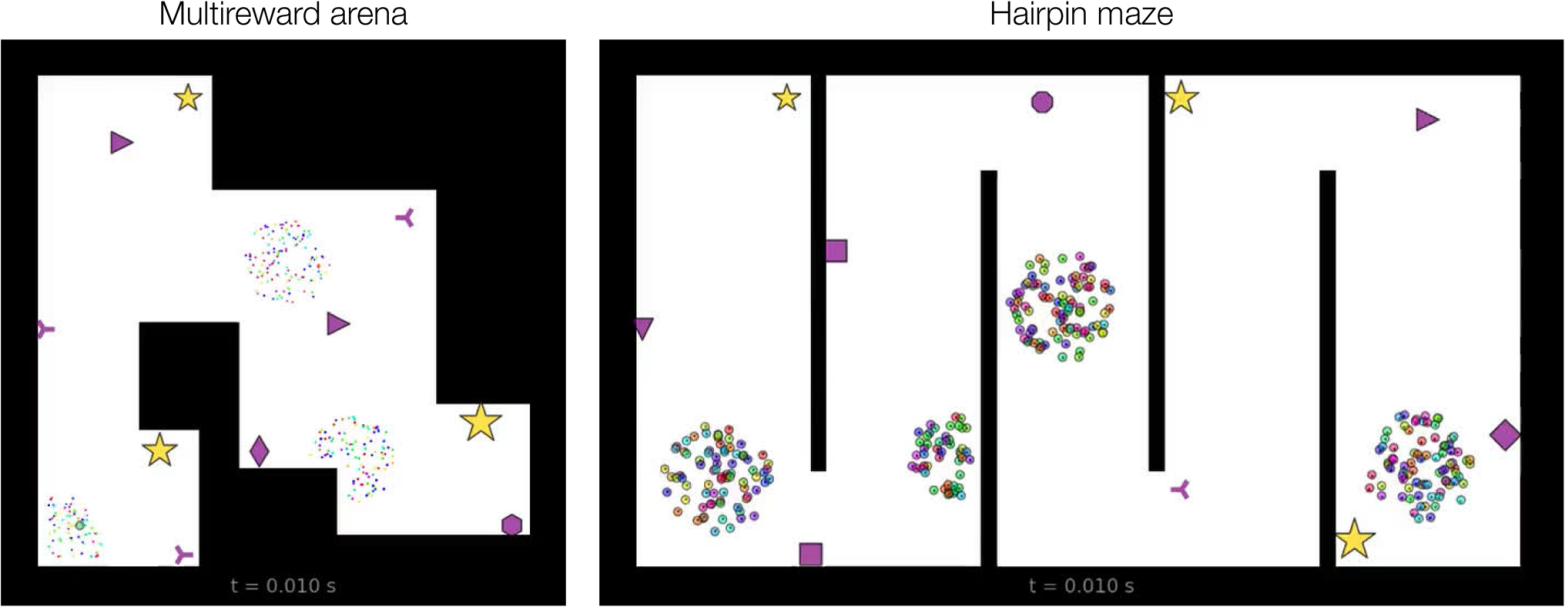
Example post-initialization ($t=0.01$ s) swarm states for NeuroSwarms simulations. (Left) A single-agent simulation in the ‘multi-reward’ arena, which contains 3 rewards (gold stars; northwest, southwest, southeast), 7 cues (purple shapes), and 3 circular regions, referred to as ‘spawn disks,’ in which random initial locations are chosen for the agents. White enclosed areas constitute the set of allowable locations for swarm agents; black regions constitute barriers and disallowed locations. Initial particle positions are sampled from the spawn disks and initial phases are random. Green circle in southwest: the single agent; dots: 300 virtual swarm particles with internal phase state indicated by color. (Right) A multi-agent simulation in the ‘hairpin’ maze, which contains 5 connected hallways, 3 rewards, 7 cues, and 4 spawn disks. Circles: 300 swarm agents with internal phase state indicated by color; reward (gold star) size is for visual differentiation only and has no effect in the model

### NeuroSwarms motion control: closing the loop

To transform updated weights into swarm motion, the NeuroSwarms controller attempts to drive the agents’ kinematic states to the equivalent desired inter-agent distances, in effect replacing the typical attraction and repulsion fields of conventional swarming models (e.g., Gazi and Passino 2011). The weights ${\varvec{W}}^{\prime }$ and ${\varvec{W}}^{r\prime }$ can be converted to desired distances ${\varvec{D}}^{\prime }$ and ${\varvec{D}}^{r\prime }$ by inverting the Gaussian swarming kernel of (3),

15 $$\begin{aligned} D^\prime _{ij} = \sqrt{-2\sigma ^2\log W^\prime _{ij}} , \end{aligned}$$

and the exponential reward kernel in (4),

16 $$\begin{aligned} D^{r\,\prime }_{ij} = -\kappa \log W^{r\,\prime }_{ij} , \end{aligned}$$

respectively. To compute the resultant swarm motion, the desired positional offset of agent *i* is averaged across its visible neighbors, i.e.,

17 $$\begin{aligned} {\varvec{f}}_i = \frac{1}{2\sum _{j} V_{ij}} \sum _{j=1}^{N_s}V_{ij}\,(D^\prime _{ij} \ - D_{ij})\frac{\mathbf {x}_j-\mathbf {x}_i}{|\mathbf {x}_j-\mathbf {x}_i|} . \end{aligned}$$

Likewise, the resultant reward-oriented motion is computed as the average

18 $$\begin{aligned} {\varvec{f}}^r_i = \frac{1}{\sum _k V^r_{ik}} \sum _{k=1}^{N_r} V^r_{ik}\,(D^{r^\prime }_{ik} \ - D^r_{ik})\frac{\mathbf {x}^r_k-\mathbf {x}_i}{|\mathbf {x}^r_k-\mathbf {x}_i|} . \end{aligned}$$

The net positional offset is calculated as a linear combination of the swarm- and reward-related offsets,

19 $$\begin{aligned} \Delta {\mathbf {x}} = \alpha {\varvec{f}}+(1-\alpha ){\varvec{f}}^r, \end{aligned}$$

where $\alpha =0.5$ for all simulations presented. The remaining processing of $\Delta {\mathbf {x}}$ serves to embed the foregoing dynamics within simulations of complex or irregular 2-dimensional environments. First, our example environments (Fig. 2) of $\sim$ 500-point height (for arbitrary point units) were processed for wall proximity and normal vectors for all allowable interior locations. Thus, we calculated a barrier-aware positional offset $\Delta {\mathbf {x}}^b$ as

20 $$\begin{aligned} \Delta {\mathbf {x}}^b_i = (1-\beta _{s_i})\Delta \mathbf {x}_i + \ \beta _{s_i}|\Delta {\mathbf {x}}_i|\,\mathbf {n}_{s_i} , \end{aligned}$$

for an exponential kernel ${\varvec{\beta }}_s=\exp (-{\varvec{d}}/\lambda )$ of distances ${\varvec{d}}$ to the nearest wall with a constant of $\lambda =20$ points, and the normal vectors $\mathbf {n}_s$ of the nearest wall. These offsets update the internal place-field locations $\mathbf {x}_s\leftarrow \mathbf {x}_s+\Delta {\mathbf {x}}^b$ of each swarm agent. Second, agent locations are updated based on the instantaneous velocity needed for each agent to approach their internal field locations, $\mathbf {v}_s = (\mathbf {x}_s-\mathbf {x}) / \Delta t$, which is processed through a momentum filter,

21 $$\begin{aligned} \mathbf {v}_\mu =\mu \mathbf {v}+(1-\mu )\mathbf {v}_s , \end{aligned}$$

with the actual velocity (prior to updating) $\mathbf {v}$ and coefficient $\mu$, a speed-limiting nonlinearity based on a kinetic-energy maximum $E_{\mathrm {max}}$,

22 $$\begin{aligned} \mathbf {v}_{\mathrm {max}}&= \sqrt{2E_{\mathrm {max}}/{\varvec{m}}} , \end{aligned}$$

23 $$\begin{aligned} \mathbf {v}_{k_i}&= \mathbf {v}_{\mathrm {max}_i}\tanh \left( \frac{|\mathbf {v}_{\mu _i}|}{ \ \mathbf {v}_{\mathrm {max}_i}}\right) \frac{\mathbf {v}_{\mu _i}}{|\mathbf {v}_{\mu _i}|} , \end{aligned}$$

where ${\varvec{m}}$ is agent mass, and barrier awareness,

24 $$\begin{aligned} \mathbf {v}_i = (1-\beta _i)\mathbf {v}_{k_i} + \beta _i|\mathbf {v}_{k_i}|\,\mathbf {n}_i , \end{aligned}$$

for proximity ${\varvec{\beta }}$ and normal vectors $\mathbf {n}$. Finally, agent locations are updated by $\mathbf {x}\leftarrow \mathbf {x}+\mathbf {v}\Delta t$.

#### Single-entity simulations

To study NeuroSwarms behavior in the context of animal spatial cognition or single-platform robotics, our implementation allows for singleton simulations analogous to conventional neural network models of spatial navigation. With minor adjustments, NeuroSwarms can operate with a single agent (i.e., $N=1$) that owns a collection of ‘virtual’ (or ‘cognitive’) swarming particles (e.g., $N_s=300$) that guide the agent’s spatial behavior. The distributed spatial behavior of the virtual swarm provides the agent with options for constructing its path through the environment. The dynamics of the virtual swarm are as described above up to (20). An array ${\varvec{V}}^\delta \in \{0,1\}^{N_s}$ indicates which particles’ positions are visible to the agent and serves to additionally mask the learning updates in (13) and (14). To produce motion, single-agent velocity is instead calculated using a cubic-activation-weighted average of the swarm,

25 $$\begin{aligned} \mathbf {v}_s = \frac{1}{\Delta t\sum _jV_j^\delta \ p_j^3}\sum _{i=1}^{N_s}V^\delta _i p_i^3 (\mathbf {x}_{s_i}-\mathbf {x}) , \end{aligned}$$

prior to processing the environmental embedding of the agent’s motion in (21)–(24). We tested several weightings of the activation term in (25), including linear, quadratic, cubic, and higher powers. We found that linear averages produced ‘indecisive’ behavior in the single agent, in which it could not consistently follow clusters of swarm particles. Cubic averages, however, achieved diffusive exploration of the environments; further analysis is needed, but we speculate that these dynamics require sufficient compression of low-activation units toward zero. Thus, the agent computes a path toward the most highly activated of the visible swarm particles.

**Table 1:** Parameters, default values, and descriptions (with units) for the NeuroSwarms controller implementation

$\Delta t$	0.01	s, Integration time step of simulation
duration	180.0	s, Total simulation time
*N*	300	No. of physical agents (multi-agent)
*N*	1	No. of physical agents (single-entity)
$N_s$	300	No. of internal fields (multi-agent) or
		virtual particles (single-entity)
$D_\mathrm{max}^\mathrm{a}$	1.0	Max. inter-agent visibility range
$E_\mathrm{max}$	3e3	$\hbox {kg}\,\hbox {points}^2/\hbox {s}^2$, Max. kinetic energy
$\mu$	0.9	Momentum coefficient of agent motion
$m_\mathrm{multi}$	0.3	kg, Mean agent mass (multi-agent)
$m_\mathrm{single}$	3.0	kg, Agent mass (single-entity)
$\sigma ^\mathrm{a}$	1.0	Spatial scale of swarm interaction
$\kappa ^\mathrm{a}$	1.0	Spatial scale of reward interaction
$\eta$	1.0	Learning rate for swarm connections
$\eta _r$	1.0	Learning rate for reward connections
$\omega _0$	0.0	cycles/s, Baseline oscillatory frequency
$\omega _I$	1.0	cycles/s, Max. increase in oscillatory
		frequency due to neural activation
$g_c$	0.4	Gain of sensory cue inputs
$g_r$	0.2	Gain of reward inputs
$g_s$	0.4	Gain of swarming inputs
$\tau _c$	0.5	s, Time constant of sensory cue inputs
$\tau _r$	0.5	s, Time constant of reward inputs
$\tau _q$	0.1	s, Time constant of swarming inputs
$d_\mathrm{rad}$	0.0	points, Reward contact radius

${^\mathrm{a}}$These parameter values are multiplicatively scaled to the notional environment size, defined in points as the radius of a disk with the same area as the set of allowable locations in the environment’s interior

### NeuroSwarms simulations

Simulated environments (Fig. 2) contained fixed rewards and cues depicted as gold stars and purple shapes, respectively. Geometry was defined by a set of linear barrier segments (e.g., walls) that formed a closed shape defining an interior space of allowable agent positions. Simulations were initialized by setting all velocities, input signals, and activations to zero; randomly choosing internal phase states; randomly assigning agent positions to special initialization regions, termed ‘spawn disks,’ that we defined for each environment; and determining agent-cue preferences ${\varvec{V}}^{c*}$  (5) based on the cues that were visible from randomly chosen allowable locations. Random seeds were reused for simulations presented to compare parameter values, unless otherwise specified. Environments were specified as vector image files that defined reward, cue, and spawn disk locations. Unless noted, parameters were set to the default values displayed in Table 1. The python source code will be available upon reasonable request.

**Fig. 3:**
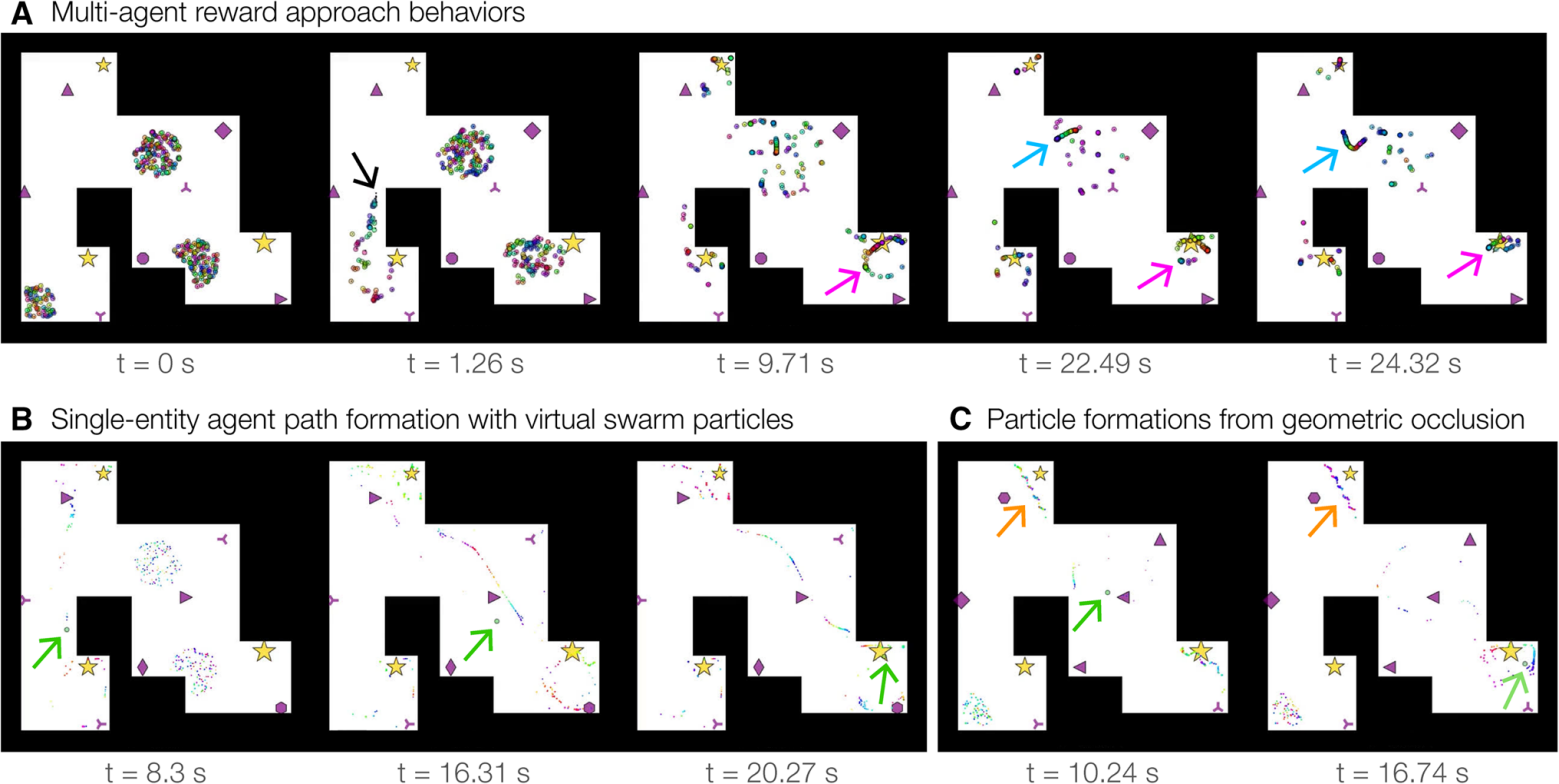
Temporal evolution of swarming and single-entity approaches to rewards. **a** Three agent clusters were initially populated in the multi-reward arena (Supplementary Video 1). The internal place-field location of each agent is indicated by a small black dot (e.g., $t=1.26$ s, black arrow). Phase sorting is indicated by sequentially ordered colors of the circle markers representing agent positions. A reward-centered phase ring was created ($t=9.71$ s) with a decreasing diameter over time ($t=22.49$ s and $t=24.32$ s; magenta arrows). A phase-sorted line segment formed and moved around a corner ($t=22.49$ s and $t=24.32$ s; blue arrows). NeuroSwarms parameters: $\sigma =1.5$, $g_c = 0.2$, $g_r=0.3$, $g_s=0.5$; Table 1. **b** A single-entity agent (larger green circle with green arrow) was guided by $N_s=300$ virtual particles (phase-colored dots; Supplementary Video 2). Swarm particles formed phase sequences leading the agent from the southwest corner to the reward location in the southeast corner of the arena by $t=20.3$ s. NeuroSwarms parameters: $\sigma =4$, $\kappa =1.5$, $g_c = 0.2$, $g_r=0.3$, $g_s=0.5$; Table 1. **c** Steplike patterns of particles (orange arrows) appeared near rewards that were occluded from the perspective of the single agent (green arrows) by corners in the environmental geometry (Supplementary Video 3). While the agent became ‘indecisive’ around $t=10.24$ s because it was pulled simultaneously in both directions, the agent ultimately found its way to the southeast reward by $t=16.74$ s. NeuroSwarms parameters: $\sigma =4$, $\kappa =8$, $g_c=0.2$, $g_r=0.3$, $g_s=0.5$; Table 1 (color figure online)

## Results

### Emergent swarming behaviors

We designed the multi-reward arena (Fig. 2, left) to characterize emergent swarming and reward-approach behaviors, and the hairpin maze (Fig. 2, right) to assess behavioral adaptability in large, fragmented environments. We observed several emergent dynamical behaviors in simulations of both multi-agent swarming and single-entity locomotion (Sect. 2, Methods). The most notable and persistent behaviors included the emergence of phase-sorted spatial formations such as line segments, rings, or concentric loops (Fig. 3; Supplementary Videos 1–3). These behaviors were analogous in form to (1) the ‘phase wave’ states observed in certain swarmalator regimes (O’Keeffe et al. 2017; see Sect. 4), and (2) the hippocampal phenomena of theta sequences and theta-rhythmic phase assemblies (Foster and Wilson 2007; Drieu et al. 2018). Further, by inspection of simulation movies, we observed two dynamical features. First, agent subgroups forming line segments and rings continuously phase-synchronized in a shared oscillation that was independent from the absolute movement or rotation of the formation in space. Second, line or ring formations would often break apart and re-form new configurations that typically involved other agents or formations that were able to phase-synchronize with elements of the subgroup. These alternating disintegrative and aggregative dynamics may be consistent with analyses of persistent homologies in place-cell networks with transient connectivity (Babichev and Dabaghian 2017).

These spatiotemporal dynamics are evident across frame captures of multi-agent (Fig. 3a; Supplementary Video 1) and single-entity (Fig. 3b; Supplementary Video 2) simulations. While phase-ordered groups could appear far from rewards (Fig. 3a, last two frames, blue arrows), swarm agents typically approached a reward location and formed a rotating ring centered on the reward position (Fig. 3a, southeast corner, last three frames). Such reward rings appeared in single-entity simulations, but the virtual swarm particles (Sect. 2.6.1) additionally exhibited particularly extended line segments that often traced out phase-ordered trajectory sequences; e.g., the agent followed an extended sequence to the reward located in the southeast corner (Fig. 3b, last two frames). Further, we observed that the size of reward rings decreased over time, reflecting a relaxation of phase and momentum given the centrally organizing reward location.

When the reward kernel’s spatial scale $\kappa$ (Table 1) was increased, streams of virtual swarm particles formed around distal rewards as the particles’ motion was modulated by agent visibility interacting with the geometry of the environment. As shown in the first frame of Fig. 3c, a steplike pattern formed near the northwest reward location while a wavy pattern formed near the southeast reward location. Both virtual swarm formations presented path choices to the single agent located in the large central compartment of the arena. As expected (Sect. 2.6.1), virtual swarm particles that were not visible to the agent remained fixed in place due to masking of the weight updates in (13) and (14). In addition to single rings, double and even triple concentric loops of nested, non-overlapping, phase-sorted rings were observed in some simulations. An example of a double loop forming is shown in the southeast corner at $t=16.74$ s (Fig. 3c; Supplementary Video 3). Strikingly, we did not adapt or tune the NeuroSwarms controller design to observe these emergent behaviors; that is, we observed parameter regimes with these behaviors upon basic implementation of several abstractions of our neuroscience-based analogies for swarming (Sects. 2.1, 2.4). Further, these behaviors would be unexpected from conventional swarming algorithms (Gazi and Passino 2011).

### Reward-based behavior in a compartmented arena

**Fig. 4:**
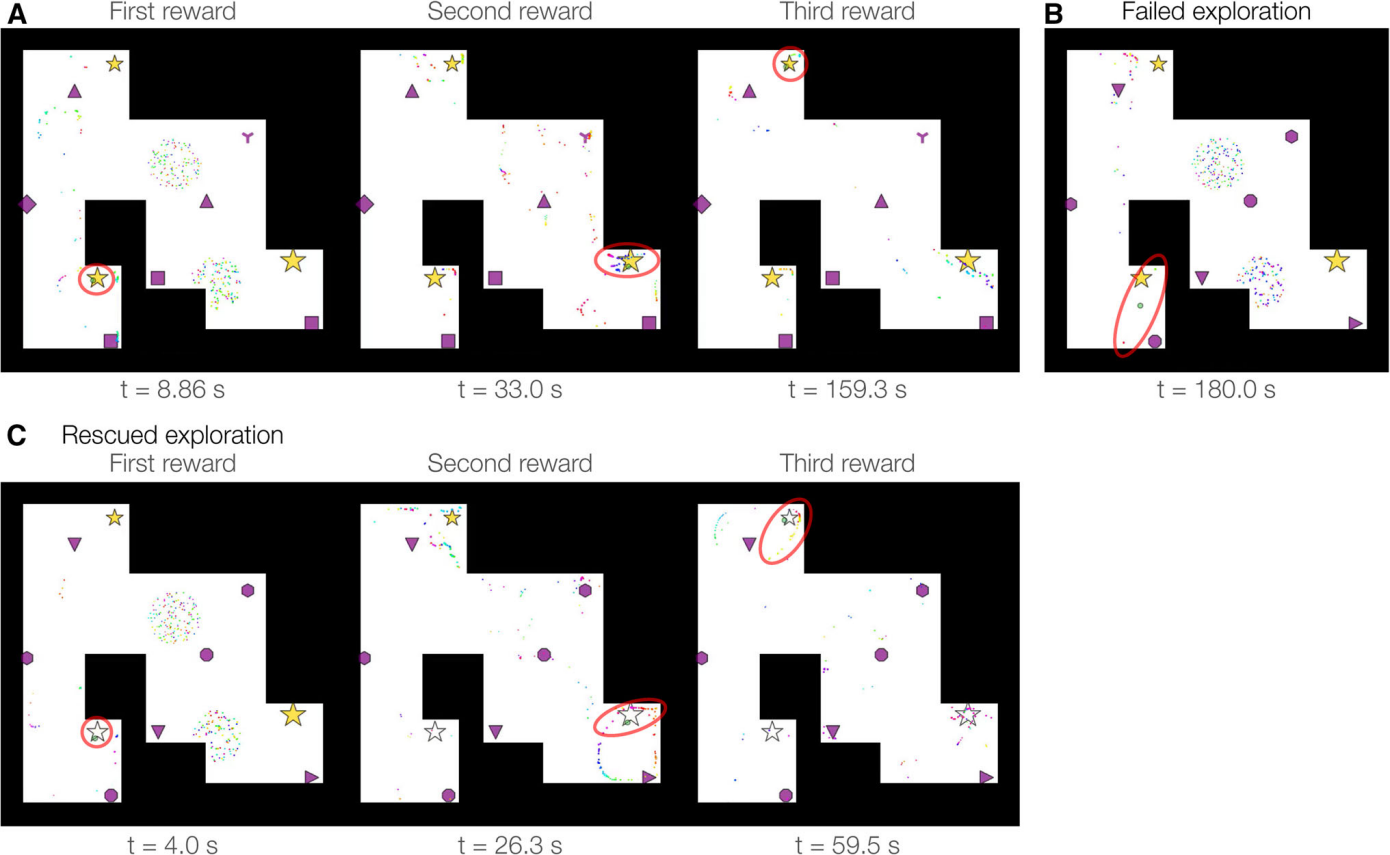
Single-entity reward-approach behavior with fixed or capturable rewards. The agent was initialized to the spawn disk in the southwest corner of the multi-reward arena. **a** A rare example in which the single agent (green circle) captured all three rewards when rewards were fixed (i.e., they remained attractive despite previous contact with the agent): southwest reward at $\sim$ 8.9 s, southeast reward at $\sim$ 33 s, and northwest reward at $\sim$ 160 s (Supplementary Video 4). Movie frames show the initial contacts with each reward (gold stars). NeuroSwarms parameters: $\sigma =4$, $\kappa =1.5$, $g_c = 0.2$, $g_r=0.3$, $g_s=0.5$; Table 1. **b** With the same parameters as (**a**) but initialized with a different random seed, this final frame of a simulation shows the converged state after the agent was attracted to the southwest corner and remained there for the duration (Supplementary Video 5). The red ellipse highlights that the agent became stuck between two fixed-point attractors that formed through mutual phase-desynchronization. **c** With the identical parameters and random seed as (**b**), rewards were made to be ‘capturable’ at a minimum contact radius of $d_\mathrm{rad}=12$ points (Sect. 2.5; Supplementary Video 6). Thus, rewards ceased to be attractive locations once the agent made initial contact. The agent captured the southwest reward at $\sim$ 5 s, the southeast reward at $\sim$ 27 s, and the northwest reward at $\sim$ 60 s. White stars indicate captured rewards. NeuroSwarms parameters: $\sigma =4$, $\kappa =1.5$, $g_c=0.2$, $g_r=0.3$, $g_s=0.5$, $d_\mathrm{rad}=12$; Table 1 (color figure online)

To assess the spatial performance of NeuroSwarms, we examined the ability of single-entity behavior to find all three rewards in the multi-reward arena. We focused on the parameter constants governing the spatial scale of swarm ($\sigma$; (3)) and reward ($\kappa$; (4)) interactions (Table 1) and found ($\sigma$, $\kappa$) values for which the agent approached multiple rewards regardless of its initial location. Due to the random initialization of location within special circular regions that we refer to as ‘spawn disks’ (Sect. 2.7), we selected 40 simulations for analysis in which the agent was spawned in the southwest corner (as in Fig. 2, left). The agent successfully captured one, two, or all three rewards in 11, 28, and 1 simulation(s) at elapsed times ranging from 4–108, 20–179, and $\sim$ 160 s, respectively. Frame captures of reward approaches are shown in Fig. 4a for the simulation in which all three rewards were found (Supplementary Video 4). The ability of the agent to approach multiple fixed rewards over time was an emergent behavior: based on our NeuroSwarms implementation, we had predicted that the rewards would serve as stable attractors in the absence of additional mechanisms such as adaptation or reward learning. Indeed, we also observed simulations which failed to explore much of the environment after approaching a single reward location. For the same parameters but a different random seed than shown in Fig. 4a, a failed exploration occurred (Fig. 4b; Supplementary Video 5) when the virtual particles split into two fixed-point, out-of-phase attractors that appeared to trap the agent.

To counter these unsuccessful equilibria, we added a ‘reward capture’ mechanism to the NeuroSwarms controller based on a minimum contact radius, $d_\mathrm{rad}$ (Sect. 2.5; Table 1). This feature causes rewards to cease being attractive locations to the virtual swarm particles upon contact by the agent, thus releasing the agent from reward-related attractors before further exploration is prevented. Indeed, having capturable rewards with $d_\mathrm{rad}=12$ points enabled a simulation that was otherwise identical to the failed case (Fig. 4b) to successfully navigate the arena to capture all three rewards (Fig. 4c; Supplementary Video 6). Thus, a notion of reward adaptation or reward consumption may be crucial to achieving continuous exploration.

**Fig. 5:**
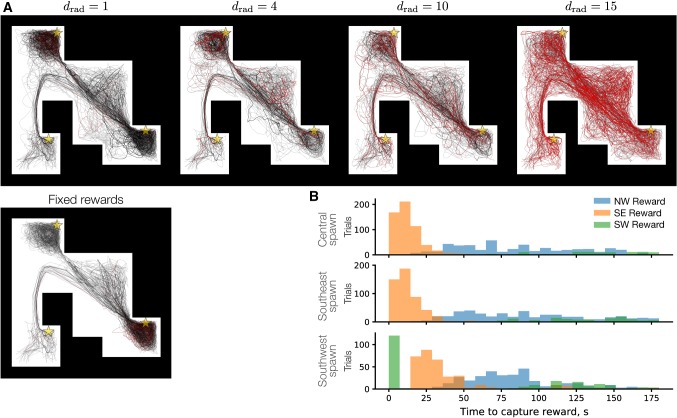
Dispersion of exploratory trajectories with capturable rewards. **a** Superimposed agent trajectories are shown from 40 single-entity simulations of 180 s duration in which the agent was initialized to the southwest corner (Sect. 2.6.1). With fixed (non-capturable) rewards, only 1 simulation (bottom, red trace) contacted all three rewards in the arena (see Fig. 4a) and there was minimal variance in the exploratory paths taken by the agent in the other simulations (black traces). The dense sampling of the northwest and southeast reward location indicates these were strong attractors for the agent. With increasing contact radii of 1, 4, 10, or 15 points (top), exploratory variance increased, the reward attractors became relatively weaker, and higher proportions of agent trajectories successfully visited all three rewards (red traces). NeuroSwarms parameters: $\sigma =4$, $\kappa =1.5$, $g_c=0.2$, $g_r=0.3$, $g_s=0.5$. Gold stars: reward locations. **b** For 700 single-entity simulations with random initial agent locations and $d_\mathrm{rad}=15$, histograms for each of the agent spawn locations (central, southeast, or southwest) display the time-to-capture profile of each of the three rewards. NeuroSwarms parameters same as the top right panel of (**a**) (color figure online)

For the 40 single-entity simulations with fixed rewards, the bottom panel of Fig. 5a reveals strong attractors at the southeast and northwest corners of the arena associated with reward locations. To demonstrate the effect of the contact radius on exploration when rewards were capturable, the trajectories resulting from contact radii of 1, 4, 10, and 15 points are shown in the top row of Fig. 5a; these values produced 1, 3, 8, and 30 (out of 40) simulated trajectories, respectively, that successfully contacted all three rewards (Fig. 5a, red traces). In a few simulations, the single-entity agent spawned in the southwest corner, found the southeast reward first, and then later returned to the southwest corner in order to collect all three rewards; such a wandering trajectory suggests that the model might qualify as an ergodic system under these conditions, but that hypothesis would be appropriately addressed by future analytical studies. These results demonstrate that the sensitivity of reward capture modulates exploratory variability by mitigating the effect of reward-related attractors. Histograms of the time-to-capture profile across agent spawn sites and reward locations reflect the structure of the environment as well as the different possible sequences of reward contact (Fig. 5b). Thus, the contact radius for capturable rewards exerted substantial control over the likelihood of the single-entity agent finding all rewards in the environment.

### Behavioral reorganization in large hairpin mazes

A key challenge for swarm controllers is adapting online to dynamic environmental changes. We consider that a multi-corridor hairpin maze encompasses adjacent spaces with potentially dissimilar properties (e.g., no reward vs. reward). Thus, as a proxy for dynamic environments, we examined adaptive behavioral changes for clusters of agents traversing hallways in a hairpin maze. This behavioral reorganization can be assessed by whether agents that spawned into no-reward hallways can nonetheless switch from random exploration to reward approach as they travel to rewarded hallways.

**Fig. 6:**
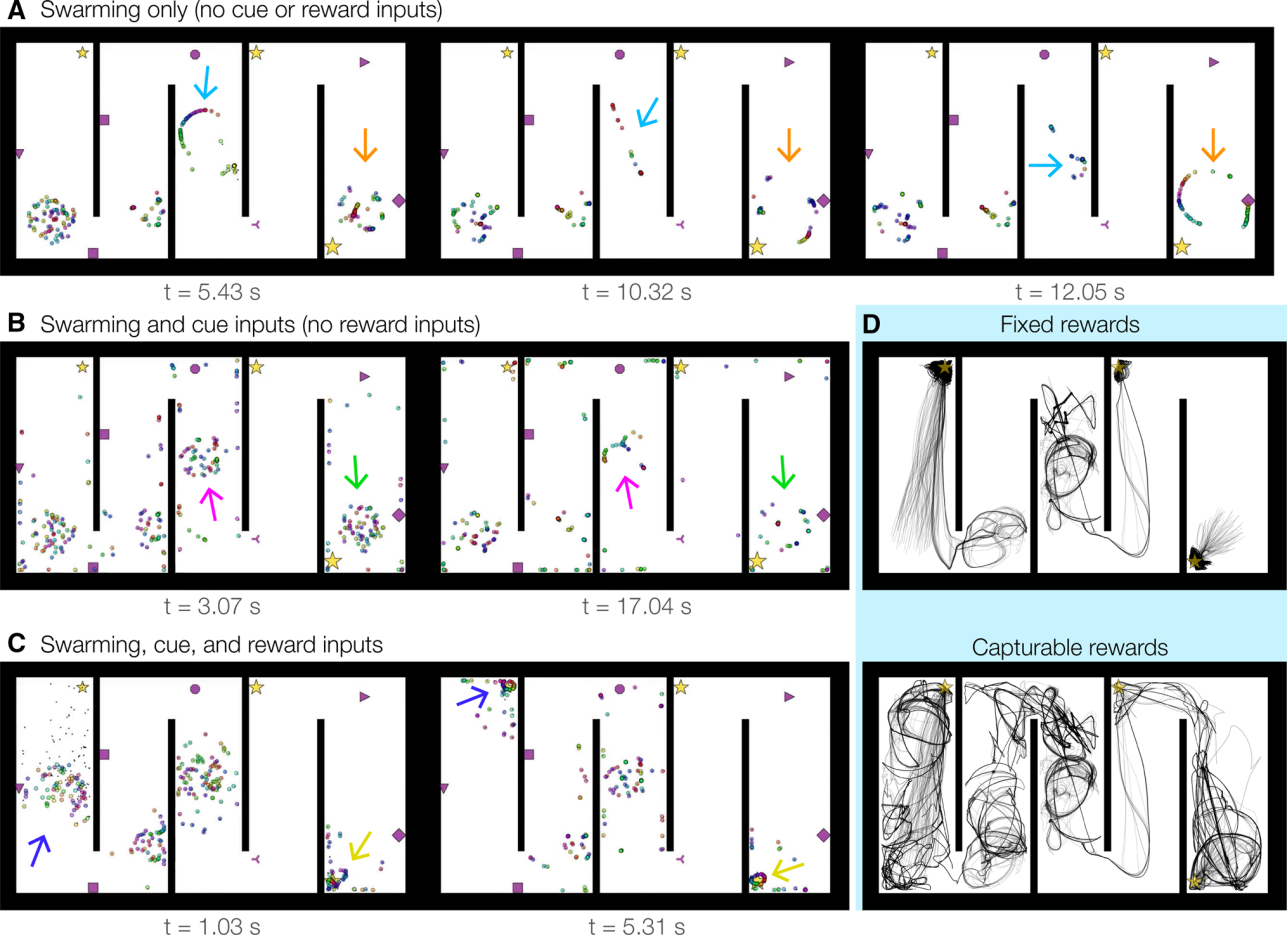
Dynamics of a multi-agent swarm in a large hairpin maze. Example frames are shown for simulations with $N=300$ agents in a rectangular environment ($885\times 519$ points including borders) partitioned into 5 hallways in a hairpin pattern. Three hallways contain rewards which are substantially occluded from the other maze sections. Emergent formations are indicated by arrows. **a** Frames from a pure swarming simulation, without reward or sensory cue influence (Supplementary Video 7). NeuroSwarms parameters: $D_\mathrm{max}=1.5$, $\sigma =2$, $\kappa =6.6$, $\eta =1$, $\eta _r=0$, $g_c=0$, $g_r=0$, $g_s=1$; Table 1. **b** Frames from a simulation with 1:1 swarm/cue input gains but no reward influence (Supplementary Video 8). NeuroSwarms parameters: $D_\mathrm{max}=1.5$, $\sigma =2$, $\kappa =6.6$, $\eta =1$, $\eta _r=0$, $g_c=0.5$, $g_r=0$, $g_s=0.5$; Table 1. **c** Frames from a simulation with equalized swarm, reward and cue input gains (Supplementary Video 9). NeuroSwarms parameters: $D_\mathrm{max}=1.5$, $\sigma =2$, $\kappa =6.6$, $\eta _s=1$, $\eta _r=1$, $g_c=g_r=g_s=1/3$; Table 1. **d** Multi-agent trajectories are shown from two 80 s simulations: fixed rewards (top) and capturable rewards with $d_\mathrm{rad}=10$ points (bottom). Compare with multi-reward arena simulations in Fig. 5a. NeuroSwarms parameters: $D_\mathrm{max}=1.5$, $\sigma =2$, $\kappa =6.6$, $g_c=0.1$, $g_r=0.1$, $g_s=0.8$; Table 1

We examined multi-agent swarming dynamics in the hairpin maze under several conditions: pure swarming (Fig. 6a; Supplementary Video 7); swarming with sensory cue inputs (Fig. 6b; Supplementary Video 8); and swarming with sensory cue inputs and reward approach (Fig. 6c; Supplementary Video 9). The sample frames shown in Fig. 6 demonstrate the emergence of phase-ordered structures in each of these conditions with the clear distinction that tightly configured reward rings became prevalent when reward inputs were activated (Fig. 6c). In that condition, it was clear that agents in the second and third hallways had difficulty navigating to other hallways with rewards. We hypothesized that this was due to the parity of swarming and reward input gains, which perhaps overemphasized reward approach at the cost of exploration in highly partitioned environments. Thus, we simulated this condition with a bias for recurrent swarming input (i.e., $g_s$ in (10)) for both fixed rewards and capturable rewards with contact radius $d_\mathrm{rad}=10$ points. Multi-agent trajectories for this enhanced exploratory regime are shown in Fig. 6d: With fixed rewards (top), the reward attractors dominate the dynamics and agents generally stayed within their initial hallways; with capturable rewards (bottom), there was substantial path variability across agents, spatial coverage increased (cf. the spiral patterns characteristic of agents’ exits from reward locations after contact), and more agents were able to traverse one hallway to the next.

**Fig. 7:**
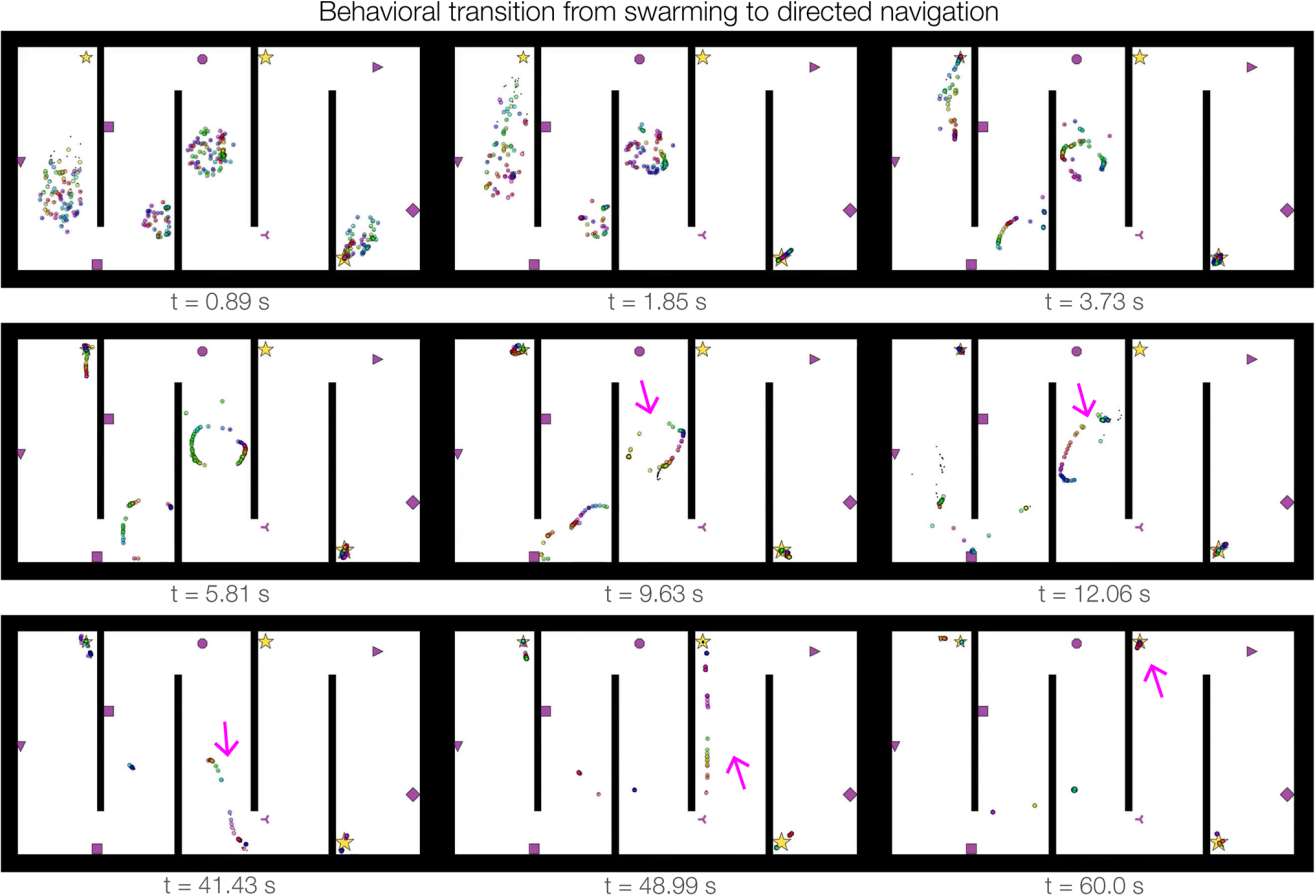
Behavioral adaptability of multi-agent swarming in the hairpin maze. Across the first 60 s of simulation (frames are shown from Supplementary Video 10), a local cluster that initialized in a corridor without rewards transitioned from random swarming behaviors to directed navigation (magenta arrows). The transition occurred when agents passed a corner into line-of-sight of the reward in the adjacent corridor (between $t=41.43$ and $t=48.99$ s). Exploratory ring formations were driven by cue heterogeneity and swarming, whereas directed trajectory sequences were oriented by reward approach. NeuroSwarms parameters: $\mathrm {duration}=300.0$, $D_\mathrm{max}=1.5$, $\sigma =2$, $\kappa =6.6$, $g_c=0.1$, $g_r=0.1$, $g_s=0.8$; Table 1 (color figure online)

To assess the converged state of multi-agent dynamics in the hairpin maze, we simulated $N=300$ agents for 300 s using the same parameters and fixed rewards as the top panel of Fig. 6d. Supplementary Video 10 shows the first 60 s of the simulation. The temporal progression of swarm state across the simulation frames presented in Fig. 7 shows distinct stages exhibited by the four initial clusters of the swarm. The two clusters that spawned in reward-free hallways eventually found their way around the barriers to adjacent hallways after milling in various line segment or ring formations for nearly a minute (Fig. 7). All of the clusters successfully converged onto the three reward locations in the maze, but the two that traversed hallways left some agents behind. The progression of those swarm clusters from initial positions to ring/arc formations to linear trajectory sequences to fixed-point reward attractors illustrates a high degree of spontaneous adaptation to the circumstances in the hairpin maze. These dynamics were self-organized and emergent, providing behaviors that resulted in nearly complete convergence to reward locations. Thus, NeuroSwarms demonstrated autonomous spatial navigation to unknown, occluded, and remote rewards in a large and complex environment.

## Discussion

We introduced the NeuroSwarms framework and an example model for studying neural control approaches to artificial autonomous swarming. We presented behaviors responding to environmental complexities such as multiple reward sites, remembered rewards, heterogeneous preferences for spatial sensory cues, and geometric constraints on visibility of cues, rewards, and other agents. We demonstrated that NeuroSwarms bridges artificial systems and theoretical models of animal spatial cognition. This reciprocity arises due to a single-entity paradigm in which the spatial behavior of a single agent is guided by an internal swarm of virtual, or ‘cognitive,’ particles. Both modes, multi-agent and single-entity, share the same underlying neural mechanisms (Sects. 2.4–2.6.1). Thus, advances in artificial systems may inform neurobiological theories of spatial cognition in large or complex environments.

### Theoretical integration of neural dynamics and artificial swarming systems

Hebbian learning in neural network models typically increments or decrements a synaptic weight according to a learning rate and a measure of the activity correlation between the presynaptic (input) and the postsynaptic (output) neurons (Hebb 1949; Levy and Steward 1979; Oja 1982; Eichenbaum 2018). For the NeuroSwarms controller, the conceptual similarity of the synaptic strength relation in a neural network and the physical distance relation in a multi-agent group allowed construction of a neural activation and learning model for the motion of artificial mobile agents. The single-entity paradigm of this model (Sect. 2.6.1) may support predictions for spatial learning experiments. For example, our model behavior predicts that reduced theta frequency during novelty exposure (Jeewajee et al. 2008; Penley et al. 2013) would correlate to larger trajectory-centered shifts in place-cell activity compared to familiar environments.

Swarms governed by NeuroSwarms self-organize into emergent, transitory configurations in position and phase that directly recall spatial attractor dynamics (Zhang 1996; Tsodyks 1999; Samsonovich and McNaughton 1997; Hedrick and Zhang 2016; Knierim and Zhang 2012) and sequential oscillatory phenomena (O’Keefe and Recce 1993; Foster and Wilson 2007; Drieu et al. 2018; Monaco et al. 2019a) that have been theorized to operate within hippocampal circuits. We explicitly designed NeuroSwarms to combine features of attractor maps and oscillatory computing using robust transformations (e.g., the spatial kernels of distance converted to synaptic strengths in (3) and (4)). The resulting model self-organized into a variety of dynamic spatiotemporal structures that recombined in complex patterns while supporting goal-finding navigation in our simulated environments. A weakness of the presented implementation was the use of a global, shared oscillation without allowing for noise, drift, or independent perturbations (cf. Zilli and Hasselmo 2010; Monaco et al. 2011, 2019a). A more decentralized approach might utilize resonant agent-oscillators that self-organize local oscillations depending on available information, task requirements, or context. Such bottom-up oscillations might aggregate into a global, swarm-wide oscillation under certain conditions, which should be studied in future models.

### Neural phase-organized swarming enables complex and heterogeneous behaviors

NeuroSwarms comprises several dynamical mechanisms that are distinct from previous swarming oscillator models. First, the weight normalization of the learning rules (13) and (14) is due to a quadratic activation term that divisively depresses connection strengths (Oja 1982). In place-cell network models, feedback inhibition typically serves to spread out place fields for efficient mapping (Savelli and Knierim 2010; Monaco and Abbott 2011), but NeuroSwarms utilizes this synaptic depression to drive agent repulsion due to the distance–weight equivalence (Sect. 2.4). In contrast, swarmalator models employ a pair-wise subtractive repulsion term (O’Keeffe et al. 2017, cf. their Eq. (3)), resulting in uniform repulsion and characteristic hexagonal tiling of agents in static states. This homogeneity of swarmalator configurations may preclude the emergence of complex transitory states as observed in NeuroSwarms simulations.

Second, phase synchronization in NeuroSwarms is intermediated by a process of neural activation. That is, net inputs drive activation (11), activation drives phase modulation (12), and the phase differences between agent pairs drive input levels (7). Thus, agents have both an intensity value (activation, analogous to neuronal firing rate) and a timing value (phase, analogous to spike timing, because spiking can be reduced to a phase description of the membrane voltage limit cycle). The link between activation and phase thus corresponds to the coupling between firing rate and theta phase observed in phaser cells (Monaco et al. 2019a). In NeuroSwarms, therefore, activation-driven attractor dynamics are simultaneously transduced into phase-driven spatial patterns that feed back to activation and local attractor states.

Future quantitative studies are required, but the causal loop described above may facilitate the observed complexity and transitory nature of swarming behaviors. In contrast, swarmalator models use local Kuramoto synchronization (or desynchronization) to achieve several spatial configurations (O’Keeffe et al. 2017, cf. their Eq. (4)). Swarmalator phase differences are directly coupled to agent motion, which may account for simpler dynamics in which swarmalators asymptotically approach stable dynamical states (Iwasa and Tanaka 2010; Iwasa et al. 2010; O’Keeffe et al. 2017). Thus, direct phase-coupled swarming may benefit from stable and predictable dynamics, whereas neural swarming mechanisms may enable complex spatial behaviors appropriate for navigation in uncertain environments.

### Cognitive swarming control for large-scale groups of small-scale platforms

We presented, at a high level, our integration of theoretical concepts from neuroscience as applied to general search problems of autonomous navigation in environments more complex than typical animal spatial cognition experiments. We asked whether the resulting dynamics might bridge critical gaps in existing neurally inspired and/or algorithmic approaches, such as limitations on online replanning and the need for resilient distributed communication strategies.

To leverage inertial, energetic, and cost benefits of small-scale robots, future applications of autonomous technologies may depend on coordinating large numbers of agents with minimal onboard sensing and communication resources. However, a challenge for autonomous multi-robot groups is that state-of-the-art control schemes break down as platforms are scaled down (decreasing agent resources) and the numerical size of groups is scaled up (increasing communication and coordination requirements) (Murray 2007; Hamann et al. 2016; Yang et al. 2018; Chung et al. 2018). The design of our NeuroSwarms model explored the conjecture that a similar distributed scaling problem may have been solved by the evolved neural architecture of mammalian brains. Compared to signal comprehension, signal production errors may be particularly deleterious to large-scale, distributed computations (Salahshour et al. 2019). Thus, onboard suites for future ‘cognitive swarming’ platforms based on NeuroSwarms principles should emphasize reliable transmission of low-bandwidth data packets (e.g., spikes or continuous phase signals). Low-fidelity inputs are more easily compensated by distributed processing; i.e., sensor designs should emphasize energy and cost to maximize deployment duration and swarm size.

Our demonstrations suggest that bottom-up, self-organized dynamics based on attractor maps and oscillatory computing open a novel path of inquiry into autonomous control. However, there are substantial estimation challenges related to, e.g., cue and reward signals, given the uncertainty of real-world environments. Future studies will be needed to understand how a NeuroSwarms system might address distributed computations including consensus, resetting, and map reconsolidation; such challenges might find more ‘neural’ interpretations that facilitate novel solutions.

### Conclusions

By analogizing agents and swarms to neurons and networks, we showed that a high-level neural approach to distributed autonomous control produces complex dynamics with navigational value. This analogy permitted the tools of theoretical neuroscience to be leveraged in developing a model controller of an artificial swarming system. The NeuroSwarms controller required two features to support cognitive swarming: (1) an internal phase state and (2) decoupling of physical location from internal self-localization. These features allowed spatial configurations of agents to be understood as attractor maps with a global oscillation, analogous to the auto-associative spatial memory and theta rhythm of hippocampal networks. Phase-based organization further leveraged the bottom-up versatility of phase-coupled mobile oscillators (Iwasa and Tanaka 2010; O’Keeffe et al. 2017; Monaco et al. 2019b). Our key insight, however, was that swarm motion can be interpreted as a mobile variation of Hebbian learning, given a natural translation between spatial relationships in a swarm and connectivity relationships in a neuronal network (Sects. 2.1, 2.4). Thus, theorized hippocampal phenomena such as attractor map formation and oscillatory sequence generation provide a framework for advances in decentralized swarm control and, reciprocally, the theoretical neuroscience of spatial navigation in large or complex environments.

## Electronic supplementary material

Below is the link to the electronic supplementary material.

Supplementary material 1 (mp4 1859 KB)

Supplementary material 2 (mp4 11448 KB)

Supplementary material 3 (mp4 3823 KB)

Supplementary material 4 (mp4 11402 KB)

Supplementary material 5 (mp4 11443 KB)

Supplementary material 6 (mp4 5737 KB)

Supplementary material 7 (mp4 1312 KB)

Supplementary material 8 (mp4 1251 KB)

Supplementary material 9 (mp4 1243 KB)

Supplementary material 10 (mp4 3814 KB)

## References

[CR1] Babichev A, Dabaghian Y (2017). Transient cell assembly networks encode stable spatial memories. Sci Rep.

[CR2] Balaji A, Das A, Wu Y, Huynh K, Dell’Anna FG, Indiveri G, Krichmar JL, Dutt ND, Schaafsma S, Catthoor F (2019). Mapping spiking neural networks to neuromorphic hardware. IEEE Trans Very Large Scale Integr (VLSI) Syst.

[CR3] Banino A, Barry C, Uria B, Blundell C, Lillicrap T, Mirowski P, Pritzel A, Chadwick MJ, Degris T, Modayil J (2018). Vector-based navigation using grid-like representations in artificial agents. Nature.

[CR4] Barrera A, Weitzenfeld A (2008). Biologically-inspired robot spatial cognition based on rat neurophysiological studies. Auton Robots.

[CR5] Bellmund JLS, Gärdenfors P, Moser EI, Doeller CF (2018). Navigating cognition: spatial codes for human thinking. Science.

[CR6] Burgess N (2014). The 2014 nobel prize in physiology or medicine: a spatial model for cognitive neuroscience. Neuron.

[CR7] Buzsáki G (2005). Theta rhythm of navigation: link between path integration and landmark navigation, episodic and semantic memory. Hippocampus.

[CR8] Casali G, Bush D, Jeffery K (2019). Altered neural odometry in the vertical dimension. Proc Nat Acad Sci.

[CR9] Chung SJ, Paranjape AA, Dames P, Shen S, Kumar V (2018). A survey on aerial swarm robotics. IEEE Trans Robot.

[CR10] Cueva CJ, Wei XX (2018) Emergence of grid-like representations by training recurrent neural networks to perform spatial localization. arXiv:1803.07770

[CR11] Cuperlier N, Quoy M, Gaussier P (2007). Neurobiologically inspired mobile robot navigation and planning. Front Neurorobot.

[CR12] Drieu C, Todorova R, Zugaro M (2018). Nested sequences of hippocampal assemblies during behavior support subsequent sleep replay. Science.

[CR13] Eichenbaum H (2018). Barlow versus Hebb: when is it time to abandon the notion of feature detectors and adopt the cell assembly as the unit of cognition?. Neurosci Lett.

[CR14] Fenton AA, Kao HY, Neymotin SA, Olypher A, Vayntrub Y, Lytton WW, Ludvig N (2008). Unmasking the CA1 ensemble place code by exposures to small and large environments: more place cells and multiple, irregularly arranged, and expanded place fields in the larger space. J Neurosci.

[CR15] Foster DJ (2017). Replay comes of age. Annu Rev Neurosci.

[CR16] Foster DJ, Wilson MA (2007). Hippocampal theta sequences. Hippocampus.

[CR17] Gaussier P, Banquet JP, Cuperlier N, Quoy M, Aubin L, Jacob PY, Sargolini F, Save E, Krichmar JL, Poucet B (2019). Merging information in the entorhinal cortex: what can we learn from robotics experiments and modeling?. J Exp Biol.

[CR18] Gazi V, Passino KM (2011). Swarm stability and optimization.

[CR19] Gupta AS, van der Meer MAA, Touretzky DS, Redish AD (2010). Hippocampal replay is not a simple function of experience. Neuron.

[CR20] Hamann H, Khaluf Y, Botev J, Divband Soorati M, Ferrante E, Kosak O, Montanier JM, Mostaghim S, Redpath R, Timmis J (2016). Hybrid societies: challenges and perspectives in the design of collective behavior in self-organizing systems. Front Robot AI.

[CR21] Hartley T, Lever C, Burgess N, O’Keefe J (2014). Space in the brain: how the hippocampal formation supports spatial cognition. Philos Trans R Soc Lond B Biol Sci.

[CR22] Hasselmo ME (2018) A model of cortical cognitive function using hierarchical interactions of gating matrices in internal agents coding relational representations. arXiv:1809.08203

[CR23] Hebb DO (1949). The organization of behavior: a neuropsychological theory.

[CR24] Hedrick KR, Zhang K (2016). Megamap: flexible representation of a large space embedded with nonspatial information by a hippocampal attractor network. J Neurophysiol.

[CR25] Iwasa M, Tanaka D (2010). Dimensionality of clusters in a swarm oscillator model. Phys Rev E Stat Nonlinear Soft Matter Phys.

[CR26] Iwasa M, Iida K, Tanaka D (2010). Hierarchical cluster structures in a one-dimensional swarm oscillator model. Phys Rev E Stat Nonlinear Soft Matter Phys.

[CR27] Jayakumar RP, Madhav MS, Savelli F, Blair HT, Cowan NJ, Knierim JJ (2019). Recalibration of path integration in hippocampal place cells. Nature.

[CR28] Jeewajee A, Lever C, Burton S, O’Keefe J, Burgess N (2008). Environmental novelty is signaled by reduction of the hippocampal theta frequency. Hippocampus.

[CR29] Jensen O, Lisman JE (2000). Position reconstruction from an ensemble of hippocampal place cells: contribution of theta phase coding. J Neurophysiol.

[CR30] Knierim JJ (2006). Neural representations of location outside the hippocampus. Learn Mem.

[CR31] Knierim JJ, Hamilton DA (2011). Framing spatial cognition: Neural representations of proximal and distal frames of reference and their roles in navigation. Physiol Rev.

[CR32] Knierim JJ, Zhang K (2012). Attractor dynamics of spatially correlated neural activity in the limbic system. Annu Rev Neurosci.

[CR33] Kreiser R, Cartiglia M, Martel JNP, Conradt J, Sandamirskaya Y (2018) A neuromorphic approach to path integration: a head-direction spiking neural network with vision-driven reset. In: IEEE international symposium on circuits and systems (ISCAS), pp 1–5

[CR34] Kunz L, Maidenbaum S, Chen D, Wang L, Jacobs J, Axmacher N (2019). Mesoscopic neural representations in spatial navigation. Trends Cogn Sci.

[CR35] Levy WB, Steward O (1979). Synapses as associative memory elements in the hippocampal formation. Brain Res.

[CR36] Milford M, Wyeth G (2008). Mapping a suburb with a single camera using a biologically inspired SLAM system. IEEE Trans Robot.

[CR37] Milford MJ, Wyeth GF, Prasser D (2004) RatSLAM: a hippocampal model for simultaneous localization and mapping. In: IEEE international conference on robotics and automation, 2004. Proceedings. ICRA’04. 2004, IEEE, vol 1, pp 403–408

[CR38] Milford MJ, Wiles J, Wyeth GF (2010). Solving navigational uncertainty using grid cells on robots. PLoS Comput Biol.

[CR39] Momennejad I, Otto AR, Daw ND, Norman KA (2018). Offline replay supports planning in human reinforcement learning. eLife.

[CR40] Monaco JD, Abbott LF (2011). Modular realignment of entorhinal grid cell activity as a basis for hippocampal remapping. J Neurosci.

[CR41] Monaco JD, Knierim JJ, Zhang K (2011). Sensory feedback, error correction, and remapping in a multiple oscillator model of place-cell activity. Front Comput Neurosci.

[CR42] Monaco JD, Rao G, Roth ED, Knierim JJ (2014). Attentive scanning behavior drives one-trial potentiation of hippocampal place fields. Nat Neurosci.

[CR43] Monaco JD, De Guzman RM, Blair HT, Zhang K (2019). Spatial synchronization codes from coupled rate-phase neurons. PLoS Comput Biol.

[CR44] Monaco JD, Hwang GM, Schultz KM, Zhang K (2019b) Cognitive swarming: an approach from the theoretical neuroscience of hippocampal function. In: Micro-and nanotechnology sensors, systems, and applications XI, International Society for Optics and Photonics, vol 10982, p 109822D

[CR45] Moser EI, Paulsen O (2001). New excitement in cognitive space: between place cells and spatial memory. Curr Opin Neurobiol.

[CR46] Moser EI, Kropff E, Moser MB (2008). Place cells, grid cells, and the brain’s spatial representation system. Annu Rev Neurosci.

[CR47] Murray RM (2007). Recent research in cooperative control of multivehicle systems. J Dyn Syst Meas Control.

[CR48] Nurzaman SG, Yu X, Kim Y, Iida F (2015). Goal-directed multimodal locomotion through coupling between mechanical and attractor selection dynamics. Bioinspir Biomim.

[CR49] Ocko SA, Hardcastle K, Giocomo LM, Ganguli S (2018). Emergent elasticity in the neural code for space. Proc Natl Acad Sci U S A.

[CR50] Oja E (1982). Simplified neuron model as a principal component analyzer. J Math Biol.

[CR51] O’Keefe J, Dostrovsky J (1971). The hippocampus as a spatial map: preliminary evidence from unit activity in the freely-moving rat. Brain Res.

[CR52] O’Keefe J, Nadel L (1978). The hippocampus as a cognitive map.

[CR53] O’Keefe J, Recce ML (1993). Phase relationship between hippocampal place units and the EEG theta rhythm. Hippocampus.

[CR54] O’Keeffe KP, Hong H, Strogatz SH (2017). Oscillators that sync and swarm. Nat Commun.

[CR55] Ólafsdóttir HF, Bush D, Barry C (2018). The role of hippocampal replay in memory and planning. Curr Biol.

[CR56] Penley SC, Hinman JR, Long LL, Markus EJ, Escabi MA, Chrobak JJ (2013). Novel space alters theta and gamma synchrony across the longitudinal axis of the hippocampus. Front Syst Neurosci.

[CR57] Pfeiffer BE, Foster DJ (2013). Hippocampal place-cell sequences depict future paths to remembered goals. Nature.

[CR58] Poll DB, Nguyen K, Kilpatrick ZP (2016). Sensory feedback in a bump attractor model of path integration. J Comput Neurosci.

[CR59] Poulter S, Hartley T, Lever C (2018). The neurobiology of mammalian navigation. Curr Biol.

[CR60] Rennó-Costa C, Tort ABL (2017). Place and grid cells in a loop: implications for memory function and spatial coding. J Neurosci.

[CR61] Rich PD, Liaw HP, Lee AK (2014). Large environments reveal the statistical structure governing hippocampal representations. Science.

[CR62] Salahshour M, Rouhani S, Roudi Y (2019). Phase transitions and asymmetry between signal comprehension and production in biological communication. Sci Rep.

[CR63] Samsonovich A, McNaughton BL (1997). Path integration and cognitive mapping in a continuous attractor neural network model. J Neurosci.

[CR64] Savelli F, Knierim JJ (2010). Hebbian analysis of the transformation of medial entorhinal grid-cell inputs to hippocampal place fields. J Neurophysiol.

[CR65] Savelli F, Knierim JJ (2018). AI mimics brain codes for navigation. Nature.

[CR66] Savelli F, Knierim JJ (2019). Origin and role of path integration in the cognitive representations of the hippocampus: computational insights into open questions. J Exp Biol.

[CR67] Savelli F, Yoganarasimha D, Knierim JJ (2008). Influence of boundary removal on the spatial representations of the medial entorhinal cortex. Hippocampus.

[CR68] Schiller D, Eichenbaum H, Buffalo EA, Davachi L, Foster DJ, Leutgeb S, Ranganath C (2015). Memory and space: towards an understanding of the cognitive map. J Neurosci.

[CR69] Tejera G, Llofriu M, Barrera A, Weitzenfeld A (2018). Bio-inspired robotics: a spatial cognition model integrating place cells, grid cells and head direction cells. J Intell Robot Syst.

[CR70] Tomov M, Yagati S, Kumar A, Yang W, Gershman S (2018) Discovery of hierarchical representations for efficient planning. bioRxiv 499418 10.1371/journal.pcbi.1007594 PMC7162548 32251444

[CR71] Tsodyks M (1999). Attractor neural network models of spatial maps in hippocampus. Hippocampus.

[CR72] Vanderwolf CH (1969). Hippocampal electrical activity and voluntary movement in the rat. Electroencephalogr Clin Neurophysiol.

[CR73] Wang C, Chen X, Lee H, Deshmukh SS, Yoganarasimha D, Savelli F, Knierim JJ (2018). Egocentric coding of external items in the lateral entorhinal cortex. Science.

[CR74] Yadav CK, Doreswamy Y (2017). Scale invariance in lateral head scans during spatial exploration. Phys Rev Lett.

[CR75] Yang GZ, Bellingham J, Dupont PE, Fischer P, Floridi L, Full R, Jacobstein N, Kumar V, McNutt M, Merrifield R (2018). The grand challenges of science robotics. Sci Robot.

[CR76] Yartsev MM, Ulanovsky N (2013). Representation of three-dimensional space in the hippocampus of flying bats. Science.

[CR77] Zhang K (1996). Representation of spatial orientation by the intrinsic dynamics of the head-direction cell ensemble: a theory. J Neurosci.

[CR78] Zilli EA, Hasselmo ME (2010). Coupled noisy spiking neurons as velocity-controlled oscillators in a model of grid cell spatial firing. J Neurosci.

